# Improving the fermentable sugar yields of wheat straw by high-temperature pre-hydrolysis with thermophilic enzymes of *Malbranchea cinnamomea*

**DOI:** 10.1186/s12934-020-01408-y

**Published:** 2020-07-25

**Authors:** Ning Zhu, Hongmei Jin, Xiangping Kong, Yanyun Zhu, Xiaomei Ye, Yonglan Xi, Jing Du, Bingqing Li, Menghan Lou, Ghulam Mustafa Shah

**Affiliations:** 1 Recycling Agriculture Research Center, Jiangsu Academy of Agricultural Sciences, Nanjing, 210014 China; 2 Key Laboratory of Crop and Livestock Integrated Farming, Ministry of Agriculture and Rural Affairs, Nanjing, 210014 China; 3 East China Scientific Observing and Experimental Station of Development and Utilization of Rural Renewable Energy, Ministry of Agriculture and Rural Affairs, Nanjing, 210014 China; 4 Jiangsu Collaborative Innovation Center for Solid Organic Waste Resource Utilization, Nanjing Agricultural University, Nanjing, 210095 China; 5 Department of Environmental Sciences, COMSATS University Islamabad, Vehari-Campus, Vehari, 61100 Pakistan

**Keywords:** Lignocellulose, Xylanase, *Malbranchea cinnamomea*, Secretome, Hydrolysis

## Abstract

**Background:**

Enzymatic hydrolysis is a key step in the conversion of lignocellulosic polysaccharides to fermentable sugars for the production of biofuels and high-value chemicals. However, current enzyme preparations from mesophilic fungi are deficient in their thermostability and biomass-hydrolyzing efficiency at high temperatures. Thermophilic fungi represent promising sources of thermostable and highly active enzymes for improving the biomass-to-sugar conversion process. Here we present a comprehensive study on the lignocellulosic biomass-degrading ability and enzyme system of thermophilic fungus *Malbranchea cinnamomea* N12 and the application of its enzymes in the synergistic hydrolysis of lignocellulosic biomass.

**Results:**

*Malbranchea cinnamomea* N12 was capable of utilizing untreated wheat straw to produce high levels of xylanases and efficiently degrading lignocellulose under thermophilic conditions. Temporal analysis of the wheat straw-induced secretome revealed that *M. cinnamomea* N12 successively degraded the lignocellulosic polysaccharides through sequential secretion of enzymes targeting xylan and cellulose. Xylanase-enriched cocktail from *M. cinnamomea* N12 was more active on native and alkali‑pretreated wheat straw than the commercial xylanases from *Trichoderma reesei* over temperatures ranging from 40 to 75 °C. Integration of *M. cinnamomea* N12 enzymes with the commercial cellulase preparation increased the glucose and xylose yields of alkali‑pretreated wheat straw by 32 and 166%, respectively, with pronounced effects at elevated temperature.

**Conclusions:**

This study demonstrated the remarkable xylanase-producing ability and strategy of sequential lignocellulose breakdown of *M. cinnamomea* N12. A new process for the hydrolysis of lignocellulosic biomass was proposed, comprising thermophilic enzymolysis by enzymes of *M. cinnamomea* N12 followed with mesophilic enzymolysis by commercial cellulases. Developing *M. cinnamomea* N12 as platforms for thermophilic enzyme mixture production will provide new perspectives for improved conversion yields for current biomass saccharification schemes.

## Background

Lignocellulosic biomass is an abundant resource for the industrial production of biofuels and high-value chemicals. Enzymatic hydrolysis is one of the crucial steps in the cost-effective process of biomass-to-bioproduct conversion. Further, mesophilic filamentous fungi, exemplified by *Trichoderma reesei*, are currently the most common industrial enzyme producers [1]. However, enzyme formulations derived from strains of mesophiles have limited thermotolerance. Development of fungal platforms that produce enzymes capable of working at high temperatures than the mesophilic conditions enables thermophilic saccharification, allowing shorter reaction time and less end-product inhibition [2].

Recently, interest in thermophilic fungi has increased in response to their ability to degrade lignocellulosic substrates at high temperatures. Glycoside hydrolases (GHs) characterized from thermophilic fungi have temperature optima between 55 and 80 °C [3, 4], whereas commercial enzyme preparations produced by mesophilic fungi perform optimally at 50 °C [5]. Consequently, these thermophilic enzymes break down plant polysaccharides faster than mesophilic enzymes at elevated temperatures. A variety of thermophilic fungi have been reported as potential producers of thermostable enzymes for lignocellulose breakdown [6–8]. *Myceliophthora thermophila* strain BJAMDU5 grown on rice straw under solid state fermentation produced high levels of cellulolytic and xylanolytic enzymes [9]. Secretome analysis of *M. thermophila* identified a number of carbohydrate-active enzymes (CAZymes) involved in lignocellulosic biomass degradation, including GHs, lytic polysaccharide monooxygenases (LPMO), carbohydrate esterases (CE) and polysaccharide lyases (PL) [9]. It has been shown that both xylan and xylose can induce significant cellulase production in thermophilic fungus *Thermoascus aurantiacus* [10]. Proteomic analysis showed that the high hydrolysis activity of *T. aurantiacus* enzymes is attributed to the predominance of proteins from GH families 5, 7, 10 and 61 in its secretome [11]. Wheat bran induced xylanolytic filamentous fungus *Talaromyces emersonii* to secret thermostable enzyme cocktails containing amylolytic, xylanolytic, glucanolytic, proteolytic and lipolytic enzymes [12]. To increase the cost-effectiveness of lignocellulosic biomass hydrolysis, sources of enzymes with improved thermal stability and catalytic efficiency should be exploited for the development of more efficient enzyme formulations.

*Malbranchea cinnamomea* was a saprophytic fungus frequently found in high temperature environments such as compost, in which decomposition of organic matter occurred at elevated temperatures (around 50 °C) [13]. Strains of *M. cinnamomea* have mostly been studied as source of enzymes that maintain high activities at temperatures between 60 to 80 °C [14]. Research conducted thus far on the hydrolytic enzymes of *M. cinnamomea* has been focused mainly on the characterization of enzymatic properties. Several hemicellulases from *M. cinnamomea* have been identified and characterized, including GH10 and GH11 xylanases [15, 16] and β-mannanase [17]. However, only a few attempts have been made to examine their hydrolysis performance on lignocellulosic biomass. Partial replacement of the commercial cellulase cocktail Cellic CTec2 with xylanases or LPMOs from *M. cinnamomea* resulted in improvement in the hydrolysis of alkali pretreated rice straw [16, 18]. A promoting effect on the hydrolysis of alkali pretreated biomass was also observed when secretome fractions of *M. cinnamomea* were supplemented to Cellic CTec2 [19]. It should be noted that biomass hydrolysis using *M. cinnamomea* enzymes in the published studies was performed at 50 °C, the optimum temperature for current commercial enzyme cocktails. Considering the thermophilic characteristic of enzymes from *M. cinnamomea*, it remains to be seen how the hydrolysis-enhancing ability of *M. cinnamomea* enzymes can be promoted at elevated temperatures.

In lignocellulosic biomass hemicellulose is closely crosslinked with the cellulose fibrils. Hemicellulose removal contributes to high sugar yields in lignocellulose hydrolysis by increasing the surface area and cellulose accessibility to cellulases [20]. Genome sequencing revealed that *M. cinnamomea* possesses a number of GHs acting on xylan, including GH5, 10, 11, 39, 43, 51 and 67 [21]. Transcriptomic analysis showed that GH10 and GH11 xylanases were highly upregulated during growth on wheat bran as compared to growth on glucose [21]. The diversity of xylan-degrading GHs in *M. cinnamomea* shows that it is a promising candidate for hemicellulose degradation. However, proteomic studies on the enzymatic system of *M. cinnamomea* is still needed to employ its hemicellulose-degrading enzymes in improving the hydrolysis of lignocellulosic biomass.

In this work, the dynamics of lignocellulosic biomass degradation by *M. cinnamomea* N12 over 10 days of solid state fermentation (SSF) were tracked in terms of changes in degradation preference, enzyme activity and substrate digestibility. Temporal alteration of the lignocellulolytic enzyme repertoire of *M. cinnamomea* N12 grown on wheat straw was profiled by quantitative secretome analysis. Furthermore, the synergistic cooperation of *M. cinnamomea* N12 enzymes with commercial cellulases in the hydrolysis of pretreated lignocellulosic materials was investigated through separate, simultaneous and sequential strategy.

## Methods

### Strain isolation and identification

The fungal strains used in this study were isolated from compost obtained from organic fertilizer plant in Nanjing, China. The compost was prepared from chicken manure mixed with wheat straw. For isolation of fungal strains, 1 g of compost sample was suspended in 10 ml physiological salt solution (8.5 g/l NaCl), plated onto potato dextrose agar (PDA) plates containing 0.1 g/l chloramphenicol (for suppression of bacterial growth) and incubated at 45 °C for 3 days. The single colony of isolated strains was obtained by means of streak plate. Fungal isolates thus obtained were inoculated at 10^5^ spores/ml into basal medium (tryptone 5.0 g/l, yeast extract 1.0 g/l, (NH_4_)_2_SO_4_ 2.0 g/l, NaCl 1.0 g/l, KH_2_PO_4_ 3.0 g/l, CaCl_2_·2H_2_O 0.3 g/l, MgSO_4_·7H_2_O 0.3 g/l, FeSO_4_ 0.3 g/l, pH 7.0) containing 1% wheat straw powder and incubated at 45 °C with constant agitation (150 rpm). After 5 days of cultivation culture the supernatant was collected by centrifugation at 6000 g for 15 min at 4 °C and immediately assayed for enzyme activities.

For species identification, total genomic DNA was extracted from fungal mycelium grown in potato dextrose broth (PDB), using E.Z.N.A^®^ Fungal DNA Mini Kit (Omega Bio-tek, GA, USA) following the manufacturer’s protocol. The extracted DNA was used as template to amplify the internal transcribed spacer (ITS) regions using the universal primers ITS1 (forward: 5′-TCCGTAGGTGAACCTGCGG-3′) and ITS4 (reverse: 5′-TCCTCCGCTTATTGATATGC-3′) [22]. Amplicons were sequenced and compared to NCBI NR and UNITE INSD databases using BLAST. The ITS sequence of N12 was deposited in GenBank under the accession number MN294559. Phylogenetic tree based on aligned ITSI-5.8S-ITSII sequences was constructed with MEGA7 using the Neighbor-Joining method [23]. The thermophilic fungi identified in this study included *Myceliophthora thermophila*, *Myceliophthora fergusii*, *Malbranchea cinnamomea*, *Scytalidium thermophilum*, *Thermomyces lanuginosus*, *Aspergillus fumigatus*, *Rhizomucor pusillus* and *Penicillium dupontii*.

The fungal biomass grown in PDB were measured at temperatures from 35 to 53 °C to determine the growth of *M. cinnamomea* N12 under varying temperature range. The liquid medium (125 mL) was inoculated with a spore suspension of *M. cinnamomea* N12 at 10^5^ spores/ml. Cultures were grown in baffled Erlenmeyer flasks (500 mL) at 35 to 53 °C for 7 days with shaking at 200 rpm, and harvested by centrifugation at 10,000*g* for 10 min at 4 °C. The dry weight of fungal mycelia was measured after freeze-drying.

### Solid state fermentation and cellulose digestibility assay

Wheat straw (WS) used in this study was harvested from a farm field in Nanjing, China during 2018. The straw sample was ground to pass through 20 mesh sieve. Thereafter, ground substrates were washed with distilled water and oven-dried at 50 °C till constant weight. The SSF was performed in 250 ml Erlenmeyer flasks containing 5 g of sterilized straw powder supplemented with 17.5 ml of Mandels’ salts solution (pH 6.5, 95 ml of water, 5 ml 20 × nitrate salts, and 0.1 ml 1000 × trace elements) [24]. Conidia of *M. cinnamomea* N12 grown on PDA plate for 7 days were suspended in physiological salt solution and inoculated in triplicate into test flasks at 2 × 10^6^ spores per g dry substrate. All test flasks were incubated at 45 °C for 10 days. WS powder without fungal inoculation under the same culture conditions was used as a control.

The WS residues during SSF were collected every 2 days by washing with 300 ml water, vacuum filtering and oven-drying. After collection, the samples were hydrolyzed by commercial cellulase mixture to evaluate the effect of *M. cinnamomea* N12 SSF on WS cellulose digestibility. In doing so, 1 g of WS sample was incubated with 40 mg of Celluclast 1.5L (389 FPU/g) supplemented with 75 mg of Novozyme 188 β-glucosidase (103 U/g) in 50 ml of 50 mM sodium acetate buffer (pH 6.0) at 50 °C. The concentration of glucose released in the hydrolysate was determined by Glucose Oxidase Assay [25] until it reached constant. The cellulose digestibility of the biotreated WS was calculated as the ratio of cellulose hydrolyzed (0.9 × glucose) to the theoretical cellulose available in the biotreated substrate. Control was also included which consist of WS without fungal inoculation. All experiments were conducted in triplicate.

### Extracellular protein extraction and enzyme activity assay

The SSF cultures of *M. cinnamomea* N12 in the test flasks were harvested at 24 h intervals by incubating with 50 ml of 50 mM citrate–phosphate buffer (pH 7.0) at 25 °C with agitation at 200 rpm for 2 h. The supernatants containing the soluble protein extracts were collected by centrifugation at 10,000*g* for 20 min at 4 °C and then vacuum-filtered through 0.22 μm membranes to remove suspended solid particles. The filtrates were used directly for enzyme activity assay. The enzyme activities were presented as units per gram dry substrate (U/gds). The protein extracts were dialyzed in 0.1 M sodium acetate buffer (pH 5.0) at 4 °C, concentrated using Amicon Ultra-15 Centrifugal Filter Unit (10-kDa cutoff, Merck Millipore) and freeze-dried for secretome profiling [24].

The cellulase, hemicellulase and ligninolytic activities in the cultures of *M. cinnamomea* N12 were analyzed as previously described [24]. Activities of endoglucanase and xylanase were measured by the dinitrosalicylic acid (DNS) method [26], with low-viscosity carboxymethylcellulose (CMC) and beechwood xylan as the substrates, respectively. For this purpose, the reaction mixture containing 50 μl of culture filtrate and 150 μl of 1.0% (w/v) substrate in 50 mM sodium acetate buffer (pH 5.0) was incubated at 50 °C for 10 min. The reaction was terminated by adding 50 μl of 1 M NaOH. After boiling with 150 μl DNS at 100 °C for 5 min, the amount of reducing sugar released was measured at 540 nm. One unit of enzyme activity was defined as the amount of enzyme that released 1 μmol of reducing sugars in 1 min from the substrate under the aforementioned conditions of assay. The activities of cellobiohydrolase, β-glucosidase and β-xylosidase were measured using *p*-nitrophenyl-β-d-cellobioside (*p*NPC), *p*-nitrophenyl-β-d-glucopyranoside (*p*NPG) and *p*-nitrophenyl-β-d-xylopyranoside (*p*NPX) as substrates, respectively. In the reaction system, 50 μl of culture filtrate was mixed with 50 μl of 200 mM sodium acetate buffer (pH 5.0) and 100 μl of 5 mM substrate. After incubation at 50 °C for 10 min, the reaction was terminated by adding 100 μl of 1 M Na_2_CO_3_. The amount of released *p*-nitrophenol was measured at 405 nm. One unit of enzyme activity was defined as the amount of enzyme that produced 1 μmol of *p*-nitrophenol in 1 min under the assay conditions.

Among the ligninase enzymes, lignin peroxidase (LiP) activity was measured using veratryl alcohol as the substrate in 100 mM sodium tartrate (pH 3.0). The culture filtrate (100 μl) was mixed with 40 mM veratryl alcohol before 10 mM H_2_O_2_ was added to start the reaction in a working volume of 1000 μl. The reaction was monitored by measuring changes in absorbance at 310 nm for 3 min at 30 °C. The extinction coefficient of veratryl alcohol was 9300 M^−1^ cm^−1^. Manganese peroxidase (MnP) activity was determined by monitoring oxidative dimerization of 2,6-dimethoxyphenol (2,6-DMP). The reaction mixture (1000 μl) consisted of 100 μl culture filtrate, 1 mM 2,6-DMP and 1 mM MnSO_4_ in 50 mM sodium tartrate buffer (pH 4.5). After the initiation of the reaction by adding H_2_O_2_ (100 μM), changes in absorbance at 469 nm were measured for 3 min at 30 °C. The extinction coefficient of 27,500 M^−1^ cm^−1^ was used for oxidized 2,6-DMP. Laccase (Lac) activity was determined with 2,2′-azonodi-3-ethylbenzothiazoline-6-sulfuric acid (ABTS) as the substrate in a total volume of 1000 μl. The reaction mixture contained 20 μl culture filtrate and 1 mM ABTS in 100 mM sodium acetate buffer (pH 4.5). Changes in absorbance at 436 nm within 3 min were monitored. The extinction coefficient of oxidized ABTS was 29,300 M^−1^ cm^−1^. One unit of ligninolytic activity was defined as the amount of enzyme that catalyzed the formation of 1 μmol of corresponding products in 1 min under the tested conditions.

### Time-course secretome analysis

The extracellular proteins of *M. cinnamomea* N12 cultivated on WS for 1, 6 and 10 days were used for secretome analysis. Lyophilized protein extracts were reconstituted with deionized water, and protein concentrations were determined using the Bradford Protein Assay Kit (GenStar, China) following the manufacturer’s instructions. For each secretome, 25 μg protein samples were reduced with 10 mM dithiothreitol at 56 °C for 45 min and alkylated with 55 mM iodoacetamide at room temperature for 30 min. The obtained protein samples were digested thoroughly using trypsin at 37 °C overnight. The peptides in the digest mixture were extracted with 30% acetonitrile in 0.1% formic acid for 30 min and 60% acetonitrile for 30 min. Peptide extracts in 0.1% formic acid were separated on a nanoAcquity UPLC (Waters, Milford, MA, USA) and subsequently analyzed by a Q-Exactive high-resolution mass spectrometer (Thermo Scientific, Waltham, MA, USA) as previously described [24]. Raw data were processed with Mascot Distiller 2.5 for peak picking. Mass spectrometry data were searched against the UniProt database for the order of *Onygenales* using the Mascot search algorithm. At least two unique peptides were required for each identified protein. False discovery rate (FDR) was set at 1% at the peptide and protein level. MaxQuant [27] with Andromeda as the search engine was used for label‑free quantification of extracellular proteins. A minimum of two ratio counts were required for valid protein quantification. Calculation of the protein LFQ intensity was based on MS peak area intensity of unique peptides using the built-in label-free quantification algorithm [28].

### Enzymatic hydrolysis of lignocellulosic biomass

The lignocellulosic substrates used in enzymatic hydrolysis included native WS and dilute alkali pretreated wheat straw (DA-WS). Native WS was ground to pass 60 mesh screen. DA-WS was prepared by pretreating WS using 1.0% NaOH at 121 °C for 1 h, with a solids loading of 5% (w/v) [29]. After the pretreatment, DA-WS was washed with distilled water to remove residual alkali and possible degradation byproducts followed by oven-drying. The lignocellulosic composition of WS and DA-WS was determined according to the analytical procedure formulated by NREL [30]. Briefly, 0.5 g of dry straw sample was extracted with 200 ml of ethanol at 95 °C to remove resin and pigment. Thereafter, the sample was subjected to two-step hydrolysis: firstly incubated in 3 ml 72% H_2_SO_4_ at 30 °C for 1 h, followed by a second hydrolysis with 87 ml of 4% H_2_SO_4_ at 121 °C for 45 min. The hydrolysates were filtered through porcelain filter crucibles. The concentrations of glucose and xylose in the liquid filtrates were measured by HPLC and used for the calculation of cellulose and xylan contents. Ash content was obtained by loss-in-ignition method where the oven-dried solid residues were combusted in muffle furnace at 575 °C for 3 h. The Klason lignin content was calculated by subtracting the ash content from the dried solid residues. The composition of native WS was 39, 32 and 10% of cellulose, xylan and lignin, respectively. The respective fraction in case of DA-WS were 62, 34 and 2%.

For temperature-dependent hydrolysis by *M. cinnamomea* N12 enzymes, each reaction mixture was prepared in 50 mM sodium acetate buffer (pH 6.0) at 2% (w/v) substrate loading in a working volume of 2 ml. The enzyme loading was set at 20 mg/g dry substrate. The hydrolysis temperatures ranged from 40 to 75 °C at an interval of 5 °C, including 40, 45, 50, 55, 60, 65, 70 and 75 °C. The commercial xylanase preparation Multifect Xylanase (Genencor, Palo Alto, CA, USA) was used as control. The reducing sugar released from the lignocellulosic biomass was quantified at the saccharification plateau using the DNS method.

For synergetic hydrolysis of DA-WS by the enzyme of *M. cinnamomea* N12 and commercial cellulases, three different strategies of enzyme addition were applied: separate, simultaneous and sequential hydrolysis. In separate hydrolysis, DA-WS was hydrolyzed by *M. cinnamomea* N12 enzymes (10 mg/g substrate) or Celluclast 1.5L (20 mg/g substrate) (Novozymes, Franklington, NC, USA) alone with the dry substrate of 2% (w/v) at 50 °C for 120 h. Enzyme–substrate mixture in 100 mM sodium acetate buffer (pH 5.0) was incubated in the reaction volume of 5 ml. In simultaneous hydrolysis (SIH), DA-WS was simultaneously hydrolyzed by the enzyme of *M. cinnamomea* N12 and Celluclast 1.5L (Novozymes) with the dry substrate of 2% (w/v) at 50 °C for 120 h. The loading of Celluclast 1.5L and *M. cinnamomea* N12 enzymes for simultaneous hydrolysis were 20 mg/g and 10 mg/g substrate, respectively. Both enzymes were added into the 50 mM sodium acetate buffer (pH 6.0) with a final volume of 5 ml at the same time. In sequential hydrolysis, DA-WS was firstly hydrolyzed by the enzyme of *M. cinnamomea* N12 at 65 °C for 24 h followed by the addition of Celluclast 1.5L and continuously incubated at 50 °C for 96 h. In the first 24 h of hydrolysis, enzymes of *M. cinnamomea* N12 was added at loading of 10 mg/g substrate. DA-WS loading was set at 2% (w/v) in 100 mM sodium acetate buffer (pH 5.0) in a working volume of 5 ml. After 24 h of hydrolysis by *M. cinnamomea* N12 enzymes at 60 °C, Celluclast 1.5L was added into the reaction system at loading of 20 mg/g substrate and incubated at 50 °C for 96 h. In all hydrolysis experiments, Novozyme 188 β-glucosidase was supplemented at a protein mass ratio of 0.034 to alleviate end-product inhibition. All hydrolysis experiments were conducted in triplicate.

The hydrolysis reaction was terminated by incubating the reaction mixture at 100 °C for 10 min. Thereafter, the hydrolysates were collected by centrifugation at 10,000*g* for 10 min and filtered through a 0.45 μm filter. The total contents of reducing sugars were quantified by the DNS method. Glucose and xylose concentrations in the filtrates were determined on an Agilent 1200 Series HPLC system equipped with an Aminex HPX-87H column (Bio-Rad) and Refractive Index Detector. The glucose and xylose yields of DA-WS were calculated as the ratio of glucan hydrolyzed (0.9 × glucose) and xylan hydrolyzed (0.89 × xylose) to theoretical glucan and xylan available in the substrate, respectively. The synergistic effect of *M. cinnamomea* N12 enzymes with commercial cellulases was evaluated by degree of synergism (DS), which was defined as the ratio of the glucose yield achieved with the combination of all enzymes and the total amount of glucose yield achieved with each enzyme alone.

### Statistical analysis

To compare the fungal biomass, enzyme activity, cellulose digestibility and LFQ intensity of cellulases and hemicellulases, analysis of variance (ANOVA) was conducted to determine the significant difference (*p* < 0.05) between the mean values of each parameter at each time point using SPSS 19.0 (SPSS Inc., Chicago, III, U.S.A.).

## Results and discussion

### Strain screening and identification

**Fig. 1:**
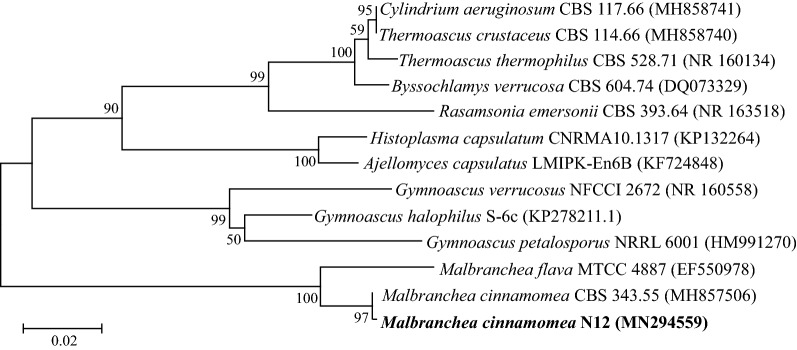
Phylogenetic analysis of *M. cinnamomea* N12 and other fungi based on ITSI-5.8S-ITSII sequence. The Neighbor-Joining (NJ) tree was constructed based on pairwise and multiple ITSI-5.8S-ITSII sequence alignment by ClustalW. Bootstrap values were obtained from 1000 replications. GenBank accession numbers of ITSI-5.8S-ITSII sequences are presented in brackets

During an extensive screening for thermophilic lignocellulolytic fungi, a total of 20 fungi capable of growing at 45 °C were isolated from composting samples. Among these fungi, strain N12 outperformed others regarding xylanase and endoglucanase activities (Additional file 1: Figure S1) and was therefore selected for further characterization. Sequence analysis of amplified internal transcribed spacer (ITS) region in NCBI NR and UNITE INSD databases revealed that the strain N12 shared a 99.85% sequence homology to that of *M. cinnamomea* CBS 343.55 (Genbank MH857506). The phylogenetic tree, constructed using neighbor-joining method based on multiple ITSI-5.8S-ITSII sequence alignment, showed that strain N12 was clustered with *M. cinnamomea* CBS 343.55 in the same clade with high bootstrap value (Fig. 1). Thus, the strain was identified as *M. cinnamomea* by its ITSI-5.8S-ITSII sequence. Best growth of *M. cinnamomea* N12 was observed at 45 °C, whereas no growth was found at temperature ≤ 35 °C or ≥ 53 °C (Additional file 2: Figure S2).

### Degradation of wheat straw by *M. cinnamomea* N12

**Fig. 2:**
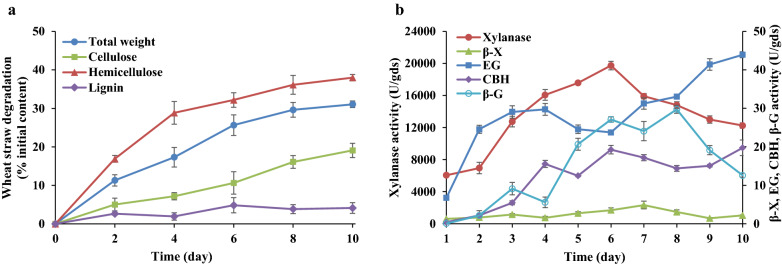
Changes in the composition of WS (**a**) and extracellular enzyme activities (**b**) during SSF with *M. cinnamomea* N12. The lignocellulosic composition of the WS residue degraded by *M. cinnamomea* N12 was determined according to the NREL laboratory analytical procedure. Total weight, cellulose, hemicellulose and lignin loss were referred to the corresponding initial content of WS. The extracellular lignocellulose-degrading enzyme activities (cellulases, hemicellulases and ligninases) in the solid culture extracts were determined every day of SSF. *β-X* β-xylosidase, *EG* endoglucanase, *CBH* cellobiohydrolase, *β-G* β-glucosidase. No lignin-degrading enzyme activities were detected during the 10-day period of SSF. The enzyme activities were presented as units per gram dry substrate (U/gds). The mean values of three replicates and standard deviations are presented

Since *M. cinnamomea* N12 was isolated from the compost pile of chicken manure mixed with wheat straw, it was specialized in producing lignocellulose-degrading enzymes using WS as the substrate. Results revealed that about 19% of the cellulose from WS was degraded during 10 days of SSF with *M. cinnamomea* N12 (Fig. 2a). The hemicellulose content decreased over the incubation period and the content of xylan in WS was 38% lower than the untreated sample, indicating the strong ability of *M. cinnamomea* N12 for breaking down the hemicellulose component of WS. During the SSF, 29% of the hemicellulose in WS was degraded during the first 4 days as compared to only 9% during the remainder period of 6 days. In contrast, the respective fractions of cellulose degradation was 7 and 12% which employed acceleration in cellulose degradation in the later stage of incubation. This might be attributed to increased accessibility of cellulose to cellulases secreted by *M. cinnamomea* N12 as a result of hemicellulosic polymer decomposition. It reflected that *M. cinnamomea* N12 utilized high xylan-degrading capacity to enable the diffusion of cellulases through the lignocellulosic matrix to interior cellulose. In comparison to polysaccharide components, lignin was hardly degraded by *M. cinnamomea* N12 as evident from Fig. 2a. The WS degradation profile revealed successive process of lignocellulose breakdown, in which hemicellulose was mainly degraded during the early stage, leading to progressively greater exposure of cellulose and increased access for secreted cellulases in the later stage.

### Secretion of lignocellulose-degrading enzymes by *M. cinnamomea* N12

In this study xylanase and cellulase activities were also detected in the solid culture extracts of *M. cinnamomea* N12 grown on WS (Fig. 2b). This corroborated with earlier studies where strains of *M. cinnamomea* have secreted high cellulolytic and hemicellulolytic activities when cultivated on a wide variety of lignocellulosic substrates, such as rice straw [29], sorghum straw [19] and wheat bran [21]. The xylanase activity in the culture extract steadily increased since incubation, reached the highest level of 19,739.0 units/g dry substrate (U/gds) on day 6 and then declined markedly thereafter. The activity of β-xylosidase remained at a low level during the first 4 days and increased to peak (4.8 U/gds) on day 7. As for cellulases, both endoglucanase and cellobiohydrolase activities increased to their maximum levels towards the end of incubation, reaching 43.9 and 19.7 U/gds, respectively. The activity of β-glucosidase increased to the peak value of 29.7 U/gds on day 8 and declined afterwards. However, the ligninolytic enzyme activities were under detectable level, which was in line with the low lignin degradation. Since *M. cinnamomea* N12 produced mainly xylanases on WS and showed the highest xylanase activity on day 6, the extracellular enzymes after 6 days of cultivation were collected and used in the following experiments.

The enzyme secretion profile showed that *M. cinnamomea* N12 had an enhanced ability to produce extracellular xylanases in the presence of WS. The level of xylanase produced by *M. cinnamomea* N12 was in line with previous reports of 27,193 (U/gds) on rice straw [31] and 24,000 U/gds on sorghum straw [19]. The high levels of xylanase activity supported *M. cinnamomea* as potent xylanase producers. Interestingly, the activity of main xylanolytic enzymes peaked on day 6 and main cellulase activities increased over the SSF, suggesting that *M. cinnamomea* N12 produced hemicellulases earlier than cellulases. The observed different patterns in the enzyme production, along with the straw degradation results revealed that *M. cinnamomea* N12 may regulate its enzyme production in response to the accessibility of polysaccharides in lignocellulosic biomass.

### Enzymatic digestibility of biotreated wheat straw

**Fig. 3:**
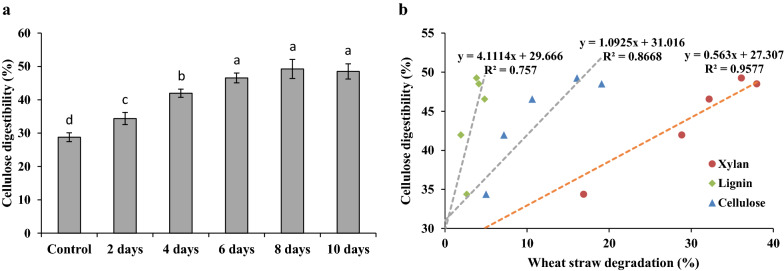
Cellulose digestibility of wheat straw degraded by *M. cinnamomea* N12 (**a**) and the correlation between cellulose digestibility and degradation extent of WS component during SSF (**b**). The cellulose digestibility of the biotreated WS was calculated as the ratio of cellulose hydrolyzed (0.9 × glucose) to the theoretical cellulose in the biotreated WS. WS without fungal inoculation was used as the control. Error bars indicate standard errors of the mean value of three replicates. Significant difference was indicated by different lowercase letters as evaluated by ANOVA at *p* < 0.05

The WS degradation results showed that *M. cinnamomea* N12 had a strong ability of degrading hemicellulose, which could be exploited for lignocellulosic biomass pretreatment. In order to evaluate the feasibility of pretreating WS with *M. cinnamomea* N12 to increase its enzymatic hydrolysis, the residual WS after SSF was hydrolyzed by commercial cellulase cocktail. The resulting WS residue after different fungal incubation time was incubated with commercial cellulase mixture at a high enzyme loading of 40 mg/g substrate at 50 °C until the glucose yields reached constant. Compared to the uninoculated WS, the cellulose digestibility of WS incubated with *M. cinnamomea* N12 was increased, which was correlated with incubation time (Fig. 3a). The maximum extent of cellulose digestibility was obtained from the straw samples after 6 days of incubation. Elongation of incubation time beyond this period could not result in further increase in glucose yield, probably due to the simultaneous degradation of cellulose component.

It was found that the hydrolysis yields of cellulose in the WS decayed by *M. cinnamomea* N12 increased nearly linearly with the xylan removal extent during SSF (Fig. 3b). A relatively higher extent of xylan solubilized in WS yielded a more pronounced increase of cellulose hydrolysis by commercial cellulases. There was no linear relationship between glucose release from the WS residues and the lignin or cellulose removal extent. The results indicated that xylanolytic enzymes secreted by *M. cinnamomea* N12 during SSF enhanced cellulose accessibility and digestibility by solubilizing xylan, presumably coating on the surface of cellulose fibers, as reported previously [32].

### Temporal dynamics of wheat straw‑induced secretome

In WS degradation assay, we observed successive stages of lignocellulosic biomass breakdown by *M. cinnamomea* N12, suggesting that the enzyme profiles may evolve in response to the changes in the chemical and structural nature of the biomass as degradation proceeded. To better understand the enzymatic machinery driving the sequential WS breakdown by *M. cinnamomea* N12, proteomic approach was employed to track the temporal alterations in the secretome of *M. cinnamomea* N12 during growth on WS. The extracellular proteins on day 1, 6 and 10 were identified using liquid chromatograph–mass spectrometry (LC–MS), and protein abundances were determined with label-free quantification by normalized label-free quantification (LFQ) intensity.

**Fig. 4:**
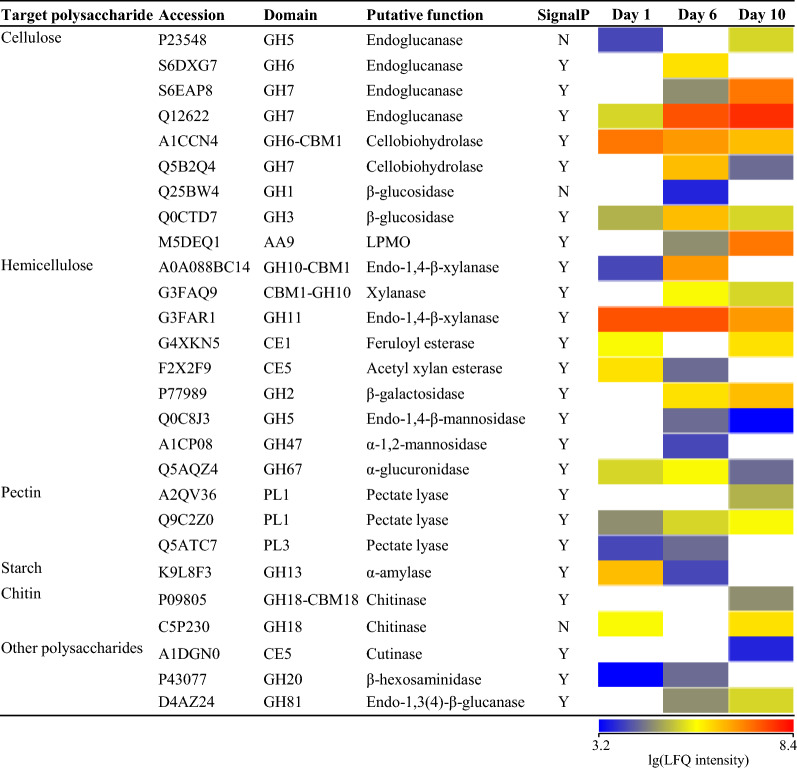
Time-course abundance of carbohydrate-active enzymes (CAZymes) in the secretome of *M. cinnamomea* N12 during growth on WS. Target polysaccharide indicated the carbohydrate substrate that CAZymes acted on. The Accession numbers and putative functions were obtained from the best hit in BLASTP against Uniprot database. Domains of CAZymes were annotated with dbCAN. Signal peptides were predicted by SignalP analysis. The abundances of CAZymes were determined by normalized label-free quantification (LFQ) intensity. The scale bar indicated lg-transformed LFQ intensities of CAZymes. Undetected CAZymes were shown in white

**Fig. 5:**
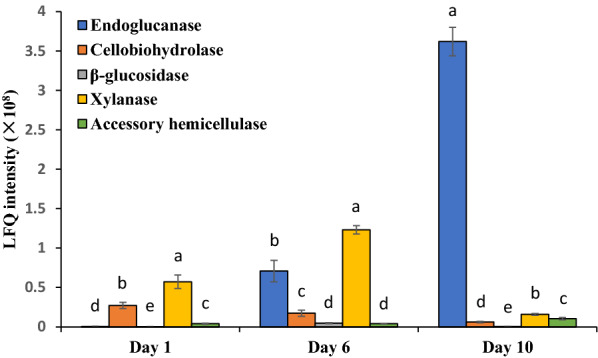
Time-course of LFQ intensity of cellulases and hemicellulases secreted by *M. cinnamomea* N12 grown on WS. Accessory hemicellulases detected in the secretome included feruloyl esterase, acetyl xylan esterase, β-galactosidase, endo-1,4-β-mannosidase, α-1,2-mannosidase and α-glucuronidase. Calculation of the protein LFQ intensity was based on MS peak area intensity of unique peptides using the MaxQuant built-in label-free quantification algorithm. Significant difference was indicated by different lowercase letters as evaluated by ANOVA at *p* < 0.05

LC–MS analysis revealed the differences in the composition of the secreted enzyme pools produced on WS over time (Fig. 4). On the first day of cultivation, 14 carbohydrate-active enzymes (CAZymes) were detected in the secretome of *M. cinnamomea* N12. A GH11 xylanase (G3FAR1) and a GH6 cellobiohydrolase with CBM1 (carbohydrate-active module family 1) (A1CCN4) were the two most abundant enzymes. Two GH5 endoglucanase (P23548 and Q12622) and a GH3 β-glucosidase (Q0CTD7) were also detected but in relatively low abundance, consistent with the comparatively low level of endoglucanase and β-glucosidase activities. Three accessory xylanolytic enzymes, feruloyl esterase (G4XKN5), acetyl xylan esterase (F2X2F9), α-glucuronidase (Q5AQZ4) were also present in the secretome on day 1. The predominance of xylan-degrading enzymes in diversity and abundance combined with the induction of high xylanolytic activity on day 1, supported that the xylan component of WS was preferentially degraded by *M. cinnamomea* N12. By day 6, a total of 21 CAZymes were represented in the secretome, including 3 endoglucanases, 2 cellobiohydrolases, 2 β-glucosidases, 1 lytic polysaccharide monooxygenase (LPMO), 3 xylanases and 5 accessory hemicellulases. GH11 xylanase (G3FAR1) remained the most abundant enzyme, followed by a GH7 endoglucanase (Q12622). By day 10, 19 CAZymes were identified in the secretome of *M. cinnamomea* N12 grown on wheat straw. The GH7 endoglucanase (Q12622) displaced GH11 xylanase (G3FAQ1) as the most abundant protein. In addition, GH7 endoglucanase (S6EAP8), AA9 LPMO (M5DEQ1) and GH6 cellobiohydrolase (A1CCN4) appeared as abundant proteins, indicating that the diversity of highly expressed cellulases may increase over time. In contrast, the abundances of all the three xylanases detected on day 6 were decreased. Figure 5 showed an obvious shift in the dominance of abundant enzymes, from xylanolytic enzymes to cellulases. Such alteration in secreted enzyme abundance was in agreement with the changes of enzyme activities in the cultures, correlating with early removal of xylan followed by attack on the consequently “unwrapped” cellulose as degradation progressed.

Fungal degradation of lignocellulosic biomass required the action of many different enzymes, which were finely orchestrated in response to the type and complexity of the encountered plant cell wall polymers [33, 34]. The sequential secretion of enzymes observed in this study unveiled the strategy employed by *M. cinnamomea* N12 to efficiently and successively break down the polysaccharides in lignocellulosic matrix. In the early stage of degradation, *M. cinnamomea* N12 mainly secreted xylanases and accessory xylanolytic enzymes to attack xylan, which was readily accessible and degradable compared to cellulose. Meanwhile, the cleavage of crystalline cellulose was initiated by secretion of cellobiohydrolases. After extensive removal of xylan, the interior cellulose chains were thereby progressively more uncovered and endoglucanases were produced in high abundance to cleave internal bonds in the cellulose chain. Xylanolytic enzymes could play a key role in initiating and potentiating lignocellulose breakdown, not only by degrading xylan, but more importantly, by creating action sites on cellulose fibers that could subsequently facilitate the action of cellobiohydrolases and endoglucanases [20]. The strategy of sequential lignocellulose breakdown by *M. cinnamomea* N12 revealed in this study inspired a new process for the hydrolysis of lignocellulosic biomass comprising enzymatic removal of xylan followed by hydrolysis with commercial cellulase preparations.

### Specific activities of different enzyme preparations on model substrates

The secretome profiling showed that the extracellular enzyme pool of *M. cinnamomea* N12 was abundant in xylanases after 6 days of cultivation on WS. Specific activities of the crude enzymes derived from *M. cinnamomea* N12 on day 6 were measured using model substrates and compared with those of commercial xylanase preparation Multifect Xylanase and commercial cellulase preparation Celluclast 1.5L, both from *T. reesei* (Table 1). Three enzyme preparations showed obvious differences in their specific activities towards model cellulosic and xylanolytic substrates. The *M. cinnamomea* N12 enzyme cocktail displayed the highest xylanase activity, indicating its high hydrolytic activity towards xylan. The xylanase activity of *M. cinnamomea* was 1.3 times higher as compared to Multifect Xylanase. In contrast, very low cellulase activities were present in the *M. cinnamomea* N12 preparation. Multifect Xylanase showed highest β-glucosidase and β-xylosidase activity among three preparations. The cellulase cocktail Celluclast 1.5L showed high endoglucanase and cellobiohydrolase activities relative to the other two enzyme preparations, while it contained low levels of β-glucosidase activity. Xylanase and β-xylosidase activities present in the Celluclast 1.5L were low, indicating its overall low hydrolysis activity towards xylan. Comparison of the enzyme activity profiles demonstrated that *M. cinnamomea* N12 enzyme preparation and Multifect Xylanase were predominant in xylanolytic activity while Celluclast 1.5L was highly active towards the cellulosic substrates.

**Table 1:** Comparison of specific activities (U/mg protein) of enzyme cocktails on model substrates

Enzyme source	EG	CBH	β-glucosidase	Xylanase	β-xylosidase
*M. cinnamomea*	0.51 ± 0.13b	0.14 ± 0.03b	0.19 ± 0.04b	77.69 ± 4.31a	0.28 ± 0.05c
Multifect Xylanase	0.31 ± 0.09c	0.07 ± 0.03c	0.26 ± 0.07a	59.17 ± 3.28b	0.61 ± 0.11a
Celluclast 1.5L	3.85 ± 0.27a	1.28 ± 0.16a	0.13 ± 0.03b	3.69 ± 0.60c	0.39 ± 0.08b

*EG* endoglucanase, *CBH* cellobiohydrolase

The mean value of three replicates are presented. Different lowercase letters indicated the significant difference (*p* < 0.05) between enzyme activities of enzyme cocktails

### Hydrolysis activity of *M. cinnamomea* N12 enzymes on lignocellulosic biomass

The enzyme activity profiling and secretome analysis indicated that the enzyme system of *M. cinnamomea* N12 cultivated on WS was featured by a wide diversity of hydrolytic enzymes, especially the enrichment of xylanase enzymes. To assess the hydrolytic potential of N12 enzymes, the hydrolysis performance of N12 enzymes harvested after 6 days of incubation was tested in comparison to the xylanase cocktail Multifect Xylanase, in the saccharification of native WS and DA-WS. Alkali pretreatment retained most of the original xylan and cellulose while removing most of lignin in the resulting materials, making it suitable for assessing the hydrolysis potential of xylanase-enriched enzyme cocktails and their synergy with cellulases.

**Fig. 6:**
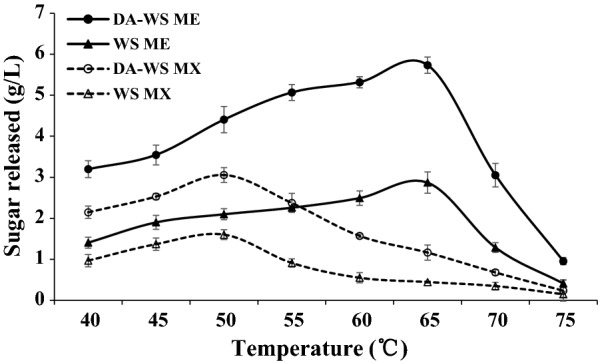
Release of reducing sugars from wheat straw by enzyme cocktail from *M. cinnamomea* N12. The extracellular enzymes from *M. cinnamomea* N12 cultured on WS for 6 days were used for hydrolysis. The hydrolysis reactions were performed with 2% (w/v) substrate loading at the temperatures indicated. Enzyme loading was at 20 mg protein/g substrate. The amount of reducing sugars released was determined using the DNS method. *WS* wheat straw, *DA-WS* dilute alkali-pretreated wheat straw, *ME M. cinnamomea* N12 enzymes, *MX* Multifest Xylanase. The mean values of three replicates and standard deviations were presented

The optimum temperature of hydrolysis for enzymes from *M. cinnamomea* N12 occurred at 65 °C, while Multifect Xylanase from *T. reesei* displayed the highest hydrolysis activity at 50 °C (Fig. 6). For each substrate tested, appreciably higher amounts of reducing sugars were released by *M. cinnamomea* N12 cocktail compared to Multifect Xylanase over a range of hydrolysis temperatures from 40 to 75 °C. The *M. cinnamomea* N12 enzyme cocktail released about twofold reducing sugar from DA-WS at 65 °C as much as that by Multifect Xylanase at 50 °C. The data in Fig. 6 indicated that the enzyme inventory of *M. cinnamomea* N12 contained mainly biomass-hydrolyzing enzymes with higher temperature optima than those of mesophilic enzyme producers like *T. reesei*.

### Synergistic hydrolysis of pretreated lignocellulosic biomass

**Fig. 7:**
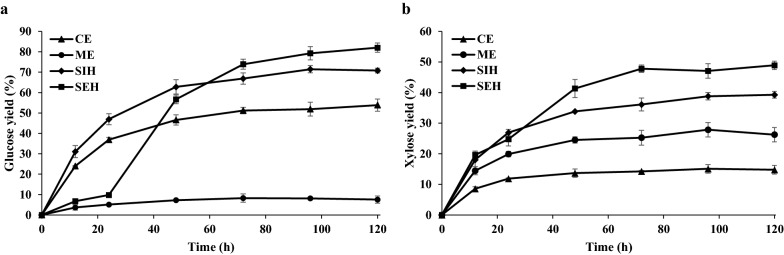
Glucose yield (**a**) and xylose yield (**b**) during hydrolysis of alkali pretreated wheat straw. The hydrolysis reactions were performed with 2% (w/v) DA-WS for 120 h. *M. cinnamomea* N12 enzymes and Celluclast 1.5L were added at 10 and 20 mg enzyme/g dry substrate, respectively. In separate hydrolysis DA-WS was hydrolyzed at 50 °C by *M. cinnamomea* enzymes (ME) or Celluclast 1.5L (CE) alone. In simultaneous hydrolysis (SIH) DA-WS was co-hydrolyzed by the enzyme of *M. cinnamomea* N12 and Celluclast 1.5L at 50 °C for 120 h. In sequential hydrolysis (SEH) DA-WS was initially hydrolyzed by *M. cinnamomea* N12 enzymes at 65 °C for 24 h and continuously incubated at 50 °C for 96 h after the addition of Celluclast 1.5L. *CE* commercial cellulases, *ME M. cinnamomea* N12 enzymes, *SIH* simultaneous hydrolysis, *SEH* sequential hydrolysis. The mean values of three replicates and standard deviations were presented

The comparison of hydrolysis activity between *M. cinnamomea* N12 enzymes and Multifest Xylanase showed that the enzyme system of *M. cinnamomea* N12 possessed catalytically efficient and thermophilic xylanase enzymes. To further analyze the ability of *M. cinnamomea* N12 enzymes to improve the hydrolysis of pretreated wheat straw by commercial cellulases, various time course hydrolysis were performed using separate, simultaneous, and sequential addition of *M. cinnamomea* N12 enzymes and commercial cellulases Celluclast 1.5L supplemented with β-glucosidase (Fig. 7). In separate hydrolysis DA-WS was hydrolyzed at 50 °C by *M. cinnamomea* enzymes (ME) or Celluclast 1.5L (CE) alone. In simultaneous hydrolysis (SIH) DA-WS was co-hydrolyzed by the enzyme of *M. cinnamomea* N12 and Celluclast 1.5L at 50 °C for 120 h. In sequential hydrolysis (SEH) DA-WS was initially hydrolyzed by the enzymes of *M. cinnamomea* N12 at 65 °C for 24 h and continuously incubated at 50 °C for 96 h after the addition of Celluclast 1.5L.

The addition of cellulases alone left nearly one half of the original cellulose in DA-WS (Fig. 7a). When *M. cinnamomea* N12 enzymes were added alone, only about 7.5% of the original cellulose was hydrolyzed into glucose. When both enzymes were added together into the hydrolysis reaction, 71% glucose yield was achieved after 120 h, whereas in the absence of *M. cinnamomea* N12 enzymes, the fraction was only 54%. Addition of *M. cinnamomea* N12 enzymes to Celluclast 1.5L resulted in substantial improvements in glucose release ranging between 27 and 38% as compared to hydrolysis with Celluclast 1.5L only. In fact, the cellulose hydrolysis yield of the combined enzyme mixture in 48 h already exceeded that by Celluclast 1.5L alone after 120 h. It was evident that the simultaneous addition of cellulases and *M. cinnamomea* N12 enzymes not only accelerated the hydrolysis rates but also increased the extent of DA-WS hydrolysis.

When the commercial cellulases were added to the *M. cinnamomea* N12 enzymes pre-hydrolyzed DA-WS (Fig. 7a), glucose yield increased sharply from 10% at 24 h of hydrolysis to about 57% at 48 h, which was significantly higher than the glucose yield achieved by the sole addition of cellulases (47% after 48 h of hydrolysis). The high temperature pre-digestion resulted in approximately 11 to 16% higher glucose yields than simultaneous hydrolysis at time points after 72 h. Besides, the hydrolysis yields of xylan were also significantly promoted by the pre-hydrolysis of *M. cinnamomea* N12 enzymes (48 vs. 39%, Fig. 7b). High temperature pre-hydrolysis step resulted in extensive xylan solubilization, which benefited subsequent mesothermal cellulose hydrolysis. A clear synergistic effect between commercial cellulases and enzymes derived from *M. cinnamomea* N12 was observed when sequential hydrolysis strategy was applied. Pre-hydrolysis at 65 °C with thermophilic enzymes from *M. cinnamomea* N12 increased the degree of synergism of cellulose and xylan conversions in DA-WS from 1.15 to 1.36 and 0.96 to 1.19, respectively.

The synergistic hydrolysis showed that regardless of the hydrolysis strategy used, the addition of *M. cinnamomea* N12 enzymes to the commercial cellulase preparation substantially increased the enzymatic digestibility of pretreated lignocellulose. This finding was consistent with a previous report that different protein fractions of *M. cinnamomea* secretome synergistically enhanced the hydrolysis of alkali pretreated carrot grass by commercial cellulase Cellic CTec2 at 50 °C [19]. In the present study the degree of hydrolysis of cellulose and xylan was higher in the sequential hydrolysis as compared to the enzymes used simultaneously. This might be due to the sub-optimum temperature where the enzyme activity of *M. cinnamomea* N12 was decreased when incubated simultaneously with Celluclast 1.5L at 50 °C, whereas the optimum temperature for the enzymes of *M. cinnamomea* N12 was 65 °C. Pre-hydrolysis at 65 °C with *M. cinnamomea* N12 enzymes resulted in higher xylan hydrolysis rate than that by mesophilic hydrolysis at 50 °C, leading to improved cellulose accessibility and digestibility despite shorter incubation time with Celluclast 1.5L.

The observed improvements in the hydrolysis yields of sequential hydrolysis were mainly attributed to high catalytic efficiency and thermostability of *M. cinnamomea* N12 xylanases that were able to maintain the activity during high temperature pre-hydrolysis step. Secretome profiling showed that in addition to thermostable xylanases, the enzyme mixture from *M. cinnamomea* N12 contained a diverse set of enzymes acting on the side chains of xylan, such as acetyl xylan esterase, feruloyl esterase, β-galactosidase and α-glucuronidase. These accessory enzymes would potentiate the hydrolysis yields of lignocellulosic biomass containing branched xylan by enhancing the action of xylanases. For instance, acetyl xylan esterases were reported to promote xylan solubilization and the subsequent hydrolysis of xylan and cellulose by removing acetyl groups from xylan [35]. Besides, the cellulases present in the enzyme mixture of *M. cinnamomea* N12 would also contribute to the boosting effect of pre-digestion on glucose yields observed in this study, as shown in the high temperature pre-hydrolysis of corn stover with thermostable endoglucanases from extremely thermophilic bacteria [36]. Secretome profiling indicated that the dominant proteins in the secretome fractions boosting the hydrolytic potential of commercial cellulases were metal dependent hydrolases, GH7 endoglucanases, monooxygenases and GH6 cellobiohydrolases, in addition to GH10 and GH11 xylanases [19]. Apart from GHs, the presence of LPMOs in the enzyme mixture of *M. cinnamomea* N12 would act synergistically with Cellulcast 1.5L through oxidative cleavage of cellulose to improve the efficiency of cellulose hydrolysis [37].

## Conclusions

This study clearly demonstrated that *M. cinnamomea* N12 was a promising source of enzymes for thermophilic lignocellulose degradation. *M. cinnamomea* N12 produced high levels of xylanases under SSF using wheat straw as the sole carbon source. Temporal secretome profiling revealed that *M. cinnamomea* N12 efficiently degraded lignocellulose through sequential secretion of carbohydrate-active enzymes. *M. cinnamomea* N12 enzymes performed better in biomass hydrolysis at high temperatures than commercial xylanases. Thermophilic pre-digestion with *M. cinnamomea* N12 enzymes resulted in better improvements in cellulose and xylan yields compared to conventional hydrolysis. This research provided a potential approach for enhancing biomass saccharification by sequential process.

## Supplementary information

**Additional file 1: Figure S1.** Comparison of xylanase and endoglucanase activities in the culture supernatants of 20 thermophilic fungi isolated in this study. Fungal culture supernatants were collected after 5 days of cultivation in basal medium containing 1% WS. Error bars indicate standard deviations from the mean value of three replicates.

**Additional file 2: Figure S2.** Growth profile of *M. cinnamomea* N12 in PDB at different temperatures. PDB was inoculated with spores of *M. cinnamomea* N12 at 10^5^ spores/ml and grown at 35-53 °C for 7 days. Fungal biomass are expressed as mycelia dry weight per ml of PDB (mg/ml). Significant difference was indicated by different lowercase letters as evaluated by ANOVA at *p* < 0.05.

## Data Availability

All data and materials described in this study are available for scientific and academic purposes upon request to the corresponding author.

## Abbreviations

CAZyme: Carbohydrate-active enzyme
CBH: Cellobiohydrolase
CBM: Carbohydrate-binding module
CE: Carbohydrate esterase
DNS: Dinitrosalicylic acid
DS: Degree of synergism
EG: Endoglucanase
FDR: False discovery rate
GH: Glycoside hydrolase
ITS: Internal transcribed spacer
LC–MS: Liquid chromatograph–mass spectrometry
LFQ: Label-free quantification
LPMO: Lytic polysaccharide monooxygenase
PL: Polysaccharide lyase
*p*NPC: *p*-Nitrophenyl-β-d-cellobioside
*p*NPG: *p*-Nitrophenyl-β-d-glucopyranoside
*p*NPX: p-Nitrophenyl-β-d-xylopyranoside
SSF: Solid state fermentation
