# Supplementary figures and images for: Improving the fermentable sugar yields of wheat straw by high-temperature pre-hydrolysis with thermophilic enzymes of Malbranchea cinnamomea
Source: Microb Cell Fact. 2020 Jul 25;19:149. doi: 10.1186/s12934-020-01408-y (PMC7382850; doi:10.1186/s12934-020-01408-y)

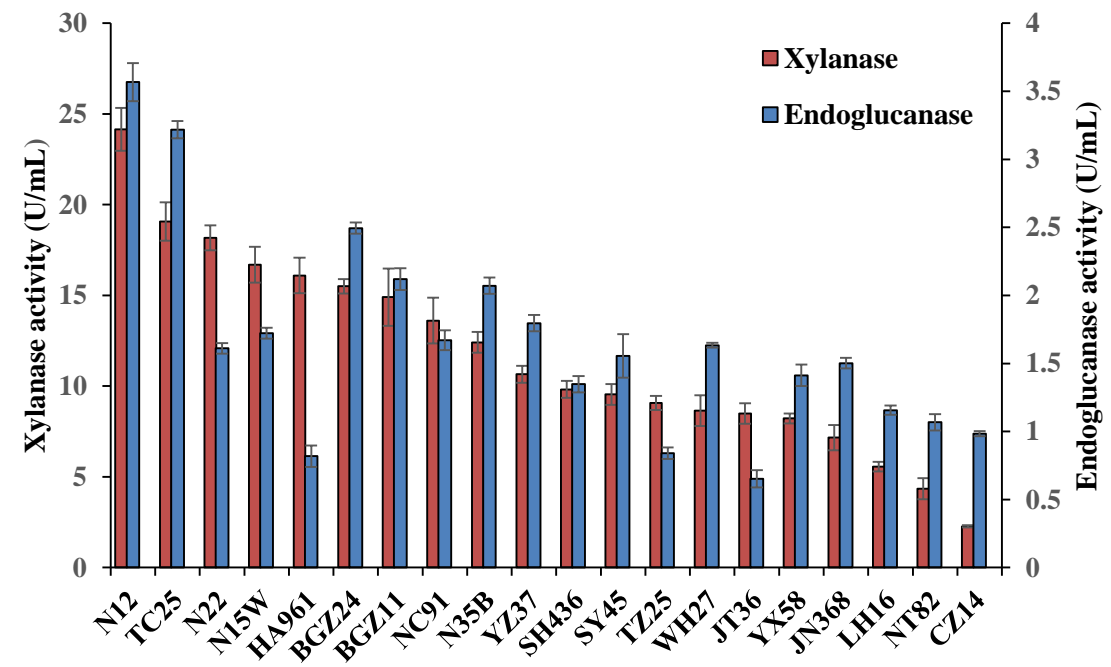

Supplement: Supplementary file 1 — Additional file 1: Figure S1. Comparison of xylanase and endoglucanase activities in the culture supernatants of 20 thermophilic fungi isolated in this study. Fungal culture supernatants were collected after 5 days of cultivation in basal medium containing 1% WS. Error bars indicate standard deviations from the mean value of three replicates. [file 12934_2020_1408_MOESM1_ESM.pdf]

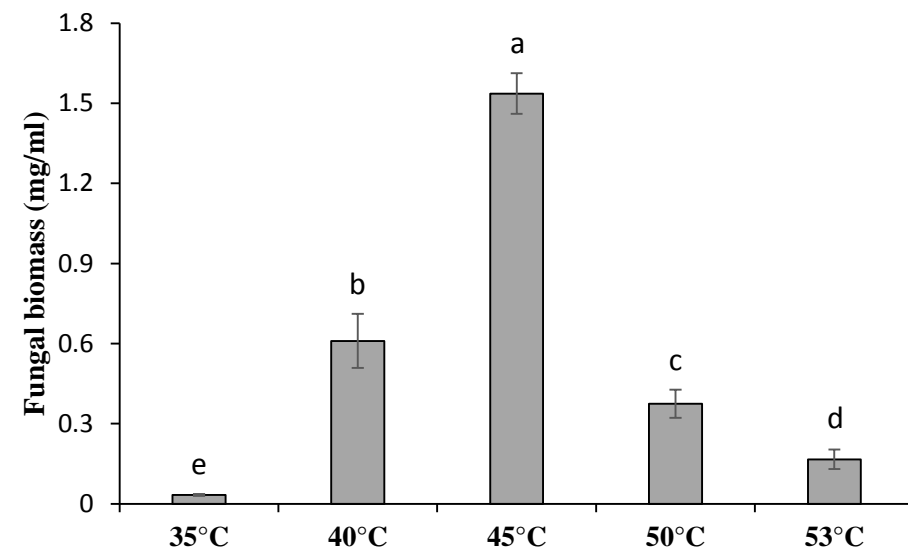

Supplement: Supplementary file 2 — Additional file 2: Figure S2. Growth profile of M. cinnamomea N12 in PDB at different temperatures. PDB was inoculated with spores of M. cinnamomea N12 at 105 spores/ml and grown at 35-53 °C for 7 days. Fungal biomass are expressed as mycelia dry weight per ml of PDB (mg/ml). Significant difference was indicated by different lowercase letters as evaluated by ANOVA at p < 0.05. [file 12934_2020_1408_MOESM2_ESM.pdf]
